# Direct Interaction of ATP7B and LC3B Proteins Suggests a Cooperative Role of Copper Transportation and Autophagy

**DOI:** 10.3390/cells10113118

**Published:** 2021-11-10

**Authors:** Supansa Pantoom, Adam Pomorski, Katharina Huth, Christina Hund, Janine Petters, Artur Krężel, Andreas Hermann, Jan Lukas

**Affiliations:** 1 Translational Neurodegeneration Section “Albrecht-Kossel”, Department of Neurology, University Medical Center Rostock, 18147 Rostock, Germany; supansapantoom@yahoo.com (S.P.); khuth@posteo.de (K.H.); Christina.Hund@web.de (C.H.); janine.petters@web.de (J.P.); Andreas.Hermann@med.uni-rostock.de (A.H.); 2 Department of Chemical Biology, Faculty of Biotechnology, University of Wrocław, 50-383 Wrocław, Poland; adam.pomorski@uwr.edu.pl (A.P.); artur.krezel@uwr.edu.pl (A.K.); 3 Center for Transdisciplinary Neurosciences Rostock (CTNR), University Medical Center Rostock, University of Rostock, 18147 Rostock, Germany; 4 German Center for Neurodegenerative Diseases (DZNE) Rostock/Greifswald, 18147 Rostock, Germany

**Keywords:** Wilson disease, ATPase copper transporting beta, autophagosome-lysosome fusion, HepG2, LC3 interaction region

## Abstract

Macroautophagy/autophagy plays an important role in cellular copper clearance. The means by which the copper metabolism and autophagy pathways interact mechanistically is vastly unexplored. Dysfunctional ATP7B, a copper-transporting ATPase, is involved in the development of monogenic Wilson disease, a disorder characterized by disturbed copper transport. Using in silico prediction, we found that ATP7B contains a number of potential binding sites for LC3, a central protein in the autophagy pathway, the so-called LC3 interaction regions (LIRs). The conserved LIR3, located at the C-terminal end of ATP7B, was found to directly interact with LC3B in vitro. Replacing the two conserved hydrophobic residues W1452 and L1455 of LIR3 significantly reduced interaction. Furthermore, autophagy was induced in normal human hepatocellular carcinoma cells (HepG2) leading to enhanced colocalization of ATP7B and LC3B on the autophagosome membranes. By contrast, HepG2 cells deficient of ATP7B (HepG2 ATP7B^−/−^) showed autophagy deficiency at elevated copper condition. This phenotype was complemented by heterologous ATP7B expression. These findings suggest a cooperative role of ATP7B and LC3B in autophagy-mediated copper clearance.

## 1. Introduction

Copper is one of the d-block metals required by several enzymes to catalyze important biochemical reactions. The major role of copper is that of a redox-active element having the ability to adopt two oxidation states, the oxidized form Cu(II) and the reduced form Cu(I) which is utilized in energy production by the respiratory chain, free radical scavenging and synthesis of neuropeptides [1,2]. The cellular copper homeostasis is tightly regulated by active ion transport across cellular membranes, which act as natural barriers, via the lysosome-associated signaling network [3]. Once copper concentration increases beyond sustainable levels, the lysosomes will undergo exocytosis to export the stored copper from cells [3,4]. The copper-transporting protein, ATP7B (Copper-transporting ATPase 2; UniProtKB–Q64446) plays a central role in copper transportation from the cytosol to the cellular organelles. Mutations of the ATP7B gene (OMIM, 606882) cause Wilson disease (OMIM, 277900), which is an inherited disorder of copper accumulation in various tissues resulting in hepatic and neurological manifestation [5]. At physiological state, i.e., normal copper level, ATP7B is a trans Golgi network (TGN) resident membrane integral protein that transfers copper from the cytosol into the lumen of the TGN [6]. Copper will be further transferred to blood circulation via copper-binding proteins such as ceruloplasmin [7]. A high intracellular copper concentration stimulates the re-localization of ATP7B to lysosomal associated vesicle compartments [4]. Lysosomal ATP7B facilitates copper uptake into the lysosomal lumen. Lysosomes containing excess copper ions will be fused with the plasma membrane to release copper into the extracellular space.

However, recent studies have shown that autophagy is also associated with the copper clearance process. First, induction of autophagy with rapamycin could reduce the copper accumulation in senescent cells and, secondly, the deletion of the copper transport protein ATP7A led to stronger copper accumulation and a more pronounced autophagy-lysosome defect [8]. A later study suggested copper-induced autophagy as a survival mechanism to prevent cell death in human hepatocellular carcinoma HepG2 cells deficient of ATP7B (HepG2 ATP7B^−/−^) [9]. The main role of autophagy is the cellular quality control. To this end, cargoes such as damaged organelles and waste products like aggregated proteins and other excess macromolecules are selectively removed [10]. The process requires double-membrane vesicles, the autophagosomes, to sequester cytosolic material prior to their fusion with endosomes or lysosomes and final degradation [11]. The autophagy process comprises induction, nucleation of the phagophore, elongation of the phagophore membrane to form the autophagosome, autophagosome-lysosome fusion and cargo degradation to produce new building blocks, and energy [12].

Autophagy induction is regulated by a number of sequential events involving different kinases. One of the major steps in autophagy is the phagophore elongation that is regulated by the formation of the lipidation of microtubule-associated protein light chain 3 (MAP1LC3, or short LC3) [13]. Lipidation of LC3 requires the cysteine protease Atg4B that processes the c-terminal end of LC3 to generate the LC3 active I variant (LC3I). Covalent conjugation of phosphatidylethanolamine (PE) to LC3I produces lipidated LC3II which in turn conjugates to phagophore membranes to promote autophagosome formation. Thus, LC3 is used as a bona fide autophagic marker [14,15].

The selection of cargoes to be degraded is mediated by the selective autophagy receptors. The receptor SQSTM/p62 (sequestosome 1) is one of the factors involved in the recognition of aggregated proteins, which are selected for elimination [16,17]. The nuclear dot protein52 (NDP52) and SQSTM/p62 receptors participate in the degradation of invading bacteria in the cellular defense mechanism, which constitutes a further vital role in which autophagy is involved [18,19]. Selective autophagy receptors are required for the recruitment of the selected cargoes on the autophagosome membranes via direct interaction of its two crucial domains, the unlipidated binding domain and the LC3 interaction region (LIR), with the ubiquitinated cargoes and LC3, respectively [20].

The LIR motifs not only play a role in the selection of the cargo to be eliminated, but LIR motifs of various proteins are also responsible for the rigorously coordinated course of the autophagy process by interaction with LC3. For instance, the LIR motifs of ULK1 are required to tether and stabilize the ULK1 complex on the autophagosome to promote the autophagy initiation and LIR motif of Atg4 is required for substrate LC3 recognition to mediate the cleavage which promotes the autophagy elongation [21,22]. Furthermore, the LIR motif has been found on the RavZ protein; the pathogenic bacteria protein from *Legionella pneumophila* that is produced to inhibit host autophagy requires the LIR motif to recognize LC3-PE for the cleavage [23,24]. This information suggests the interaction of distinct proteins containing LIR motifs with LC3 expanding interplay of autophagy and other cellular pathways.

The present study was designed to provide further mechanistic insights on whether the copper dependent ATPase ATP7B can act as an interface in the communication between unsustainably high copper concentrations and autophagy regulation. We found that one of the LIR motifs, designated LIR3, interacted with LC3B in vitro. Further evidence indicates partial colocalization of ATP7B and LC3B on the surface of the autophagosome membrane in HepG2 cells. Substitution of the conserved hydrophobic residues W1452 and L1455 on the LIR3 motif to alanine reduced the interaction of ATP7B and LC3B in vitro and in the cells. HepG2 cells deficient of ATP7B showed impaired autophagosome-lysosome fusion under elevated copper levels. Altogether, these results suggest that correct autophagy-mediated copper clearance depends on the LIR-motif mediated translocation of ATP7B to the autophagosome membrane.

## 2. Materials and Methods

### 2.1. Cell Lines, Antibodies and Subcellular Markers

HepG2 cells deficient of ATP7B (HepG2 ATP7B^−/−^) and the parental HepG2 wild-type (WT) cells were obtained from Horizon (Cambridge, UK). HEK293H cells were purchased from Invitrogen (Dreieich, Germany) (11631017).

Primary antibodies were obtained from the following sources: rabbit anti-ATP7B (Abcam, Cambridge, United Kingdom, ab124973), rabbit anti-LC3B (Cell Signaling Technology, Danvers, MA, USA, 2775s), rabbit anti SQSTM/p62 (Cell Signaling Technology, 39749), mouse anti- glyceraldehyde-3-phosphate dehydrogenase (GAPDH) (Abcam, ab8245) and rabbit anti-glutathione-S-transferase (GST) (Sigma-Aldrich, Steinheim, Germany, G7781). Secondary antibodies used for western blot detection were purchased from the following sources: Alexa Fluor 680 goat anti-mouse (Thermo Fisher Scientific, Dreieich, Germany, A-21057) and Alexa Fluor 680 goat anti-rabbit (Thermo Fisher Scientific, A-22109). Secondary antibodies used for immunofluorescence detection were purchased from the following sources: Alexa Fluor 488 goat anti-mouse (Thermo Fisher scientific, A-11029), Alexa Fluor 488 goat anti-rabbit (Thermo Fisher Scientific, A-11034), Alexa Fluor 568 goat anti-mouse (Thermo Fisher Scientific, A-21043) and Alexa Fluor 568 goat anti-rabbit (Thermo Fisher Scientific, A-11036). Autophagosome-lysosome fusion was detected using BacMam 2.0 expressing tandem sensor LC3B fused to a red fluorescent protein (RFP)–green fluorescent protein (GFP)–(Molecular Probes, Carlsbad, CA, USA, P36239). Lysosomal staining was achieved using BacMam 2.0 virus expressing lysosomal associated membrane protein 1 (LAMP1) fused to a RFP (Thermo Fisher Scientific, C10504). Trans-Golgi network visualization was obtained using BacMam 2.0 expressing N-acetylgalactosaminyltransferase (GALNT) fused to RFP (Thermo Fisher Scientific, C10593).

### 2.2. Identification of Canonical LIR Motifs on ATP7B

The LIR motifs of ATP7B were identified from the iLIR webserver [25]. The amino acid sequence of ATP7B (UniProtKB, P35670) was submitted on the server and 21 canonical LIR-motifs were identified with an internal consensus sequence of W/F/YxxL/I/V (where x can be any amino acid). Furthering filtering of the results based on the knowledge that potential LIR motifs are located on the flexible or disordered regions of protein structures identified three likely LIR motifs on the ATP7B protein, including LIR1 (FAFDNV), LIR2 (TPWDQV), and LIR3 (DKWSLL).

### 2.3. Peptide Synthesis

The three potential LIR peptides, LIR1, LIR2, LIR3 and the corresponding variants were prepared by solid-phase synthesis using the Fmoc strategy on TentaGel RAM resin essentially as described before [26]. Peptides were synthesized using a fully automated Liberty 1 microwave-assisted synthesizer (CEM). Each peptide was labelled by conjugating 5-carboxyfluorescein (CF) (Merck, 851025) to N-terminus. Peptide cleavage was achieved with a mixture of 90% trifluoroacetic acid (TFA), 5% 1,2-EDT, 3% H_2_O and 2% TIPS over 1 h, followed by precipitation in cold diethyl ether. Crude peptide was collected by centrifugation. Peptides were purified by HPLC (Dionex Ultimate 3000, Thermo Fisher Scientific, Dreieich, Germany) using a semipreparative Gemini-NX C18 column (Phenomenex, Aschaffenburg, Germany) with a gradient of acetonitrile in water with 0.1% TFA or gradient of acetonitrile in 10 mM (NH_4_)_2_CO_3_ and later lyophilized. The identity of purified peptide was confirmed by electrospray ionization-mass spectrometry using an API 2000 (Thermo Fisher Scientific) instrument. Calculated and experimental monoisotopic masses were in agreement (for details see Supplementary Table S1). Before use, the peptide was dissolved in the HEPES buffer, containing 50 mM HEPES (Carl Roth, Karlsruhe, Germany, 230-907-9) pH 7.2, 50 mM NaCl (Carl Roth, 231-598-3) and 2 mM 1,4-Dithioerythritol (DTE) (Sigma Aldrich, D8255).

### 2.4. Cloning and Purification of LC3B

LC3B gene was cloned into the expression pET-His_6_-MBP (Addgene, Watertown, MA, USA, 29708) and pET-His_6_-glutathione S-transferase (GST) (Addgene, 29655) vectors containing a TEV cleavage site located downstream from the affinity tags. The forward (TAAAATATTATGCCGTCGGAGAAGACC) and reverse primers (TAAGGATCCGTTACACTGACAATTTCATCCCGAA) containing SspI and BamHI enzyme restriction sites were used for the amplification of LC3B cDNA from pEGFP_LC3B vector was a kind gift from Karla Kirkegaard made available at addgene: http://n2t.net/addgene:11546; (accessed on 3 January 2019). The pET-His_6_-MBP-LC3B vector was used for LC3B protein production. The expression host, E. coli BL21-codonplus (DE3)-RIL (Agilent Santa Clara, CA, USA, 230245) cells were transformed with the pET-His_6_-MBP-LC3B vector. Protein expression was induced with 0.4 mM isopropyl β-D-1-thiogalactopyranoside (Carl Roth, 2316.3) at 20 °C overnight. The cells were harvested and resuspended in HEPES buffer containing 1 mg/mL lysozyme (Sigma Aldrich, L6876) and 1X protease inhibitor cocktail (Roche Life Science, Penzberg, Germany, 11697498001). Incubation at room temperature for 30 min and subsequent sonication was carried out to attain complete cell lysis. The supernatant was collected by centrifugation at 75,600× *g* using Beckman Coulter J-30I Avanti Centrifuge and JA-25.50 rotor for 30 min at 4 °C. The LC3B protein, containing His_6_/MBP tag at the N-terminus, was purified using Ni-NTA affinity chromatography (QIAGEN, Hilden, Germany, 30210). The tags were cleaved with TEV protease (The European Molecular Biology Laboratory (EMBL), Heidelberg, Germany) and removed by performing a second step of Ni-NTA affinity chromatography. Protein concentration was measured with the Pierce™ BCA Protein Assay Kit (Thermo Scientific, 23227).

### 2.5. Fluorescent Polarization

Binding experiments were done in a black 384-microwell plate. The CF-labelled peptides and purified LC3B protein were dissolved in a HEPES buffer. Each CF-labelled peptide in a volume of 25 µL at a concentration of 2.5 µM was combined with 25 µL of LC3B at a concentration range from 0.2–330 µM. The fluorescent polarization was measured with the excitation and emission wavelengths of 535 nm and 585 nm, respectively. The obtained polarization data were corrected by subtraction of the peptide alone (blank). The dissociation constant (*K*_d_) was quantified using a nonlinear curve fitting the site-specific binding on GraphPad Prism 5.0 for Windows (GraphPad Software, La Jolla, CA, USA). Experiments were performed in three independent replications.

### 2.6. Pull-Down Experiment with GST Affinity Tag

GST tagged LC3B was used as a bait to trap the full-length ATP7B from a lysate obtained from ATP7B transfected human embryonic kidney 293 H cell line (HEK293H) cells. To this end, 100,000 cells of HEK293H were cultured in 1 mL of DMEM media (Thermo Fisher Scientific, 31966-047) plus 10% fetal bovine serum (FBS) (Fisher Scientific, 10309433) and 1% Penicillin-Streptomycin (P/S) (Thermo Fisher Scientific, 15140122). Once the cells reached 80% confluence, cells were transfected with pIRES-GFP carrying full-length ATP7B (NM_000053.4) or its W1452A/L1455A mutant using Lipofectamine LTX and plus reagent (Invitrogen, 15338100). After 24 h post transfection, the HEK293H cells were lysed with RIPA buffer (10 mM Tris-HCl (Carl Roth, 214-684-5) pH 8.0, 1 mM ethylenedinitrilotetraacetic acid (Sigma Aldrich, 1084170250), 1% Triton X-100 (Sigma Aldrich, 1122980101), 0.1% sodium deoxycholate (Sigma Aldrich, D6750), 0.1% sodium dodecyl sulfate (SDS) (Carl Roth, 205-788-1), 140 mM NaCl (Carl Roth, 231-598-3) and 1 mM phenylmethylsulfonyl fluoride (Sigma Aldrich, 93482) to obtain a protein lysate. Recombinant LC3B was obtained using 10 mL of overnight expression of E. coli BL21 (DE3) cells transformed with pET-His_6_-GST-LC3B. The cells were lysed as described above and the lysate was loaded onto column containing 5 mL glutathione agarose resin (Thermo Fisher Scientific, 16100). The resin was washed with 10 column volume of HEPES buffer. Pull-down was performed using 500 µg of ATP7B containing HEK293H cell protein lysate to a volume of 200 µL of the resin bound GST tagged LC3B. The resin was washed three times with 10 column volumes of the HEPES buffer. Subsequently, elution was carried out by adding 1X Laemmli buffer (0.1% 2-mercaptoethanol, 0.0005% bromophenol blue, 10% glycerol, 2% SDS (Carl Roth, 205-788-1) in 63 mM Tris-HCl pH 6.8). The negative control experiment was done by using a GST tag as a bait. The fractions from the pull-down experiments were loaded on a Criterion TGX precast 4–15% Tris-HCl gel (Bio-Rad, 5678083) and SDS page and western blot were performed. Blots were imaged on an Infrared fluorescence imaging system using Li-Cor 9120 Odyssey (LI-COR Biosciences, Bad Homburg, Germany). Protein band intensity quantification was done using ImageJ (National Institutes of Health, Bethesda, MD, USA).

### 2.7. Autophagy Detection

5 × 10^5^ HepG2 cells were cultured in 2 mL of DMEM medium supplemented with 10% FBS and 1% P/S. Once the cell reached 80% confluence, they were treated with varied concentration of CuSO_4_ (Sigma Aldrich, 209198) from 200 µM to 800 µM for 24 h. For the starvation induction, cells were additionally treated with Earle’s Balanced Salt Solution (EBSS) media (Sigma Aldrich, E3024) for 2 h with or without 50 µM chloroquine (CLQ) prior to lysis with RIPA buffer containing inhibitor cocktails (Roche Life Science, 11697498001). Protein concentration was determined to be sure that the protein of each sample was equally loaded on the Criterion TGX precast gel for western blot detection. ATP7B, GAPDH and LC3B-II/LC3B-I was determined using the above-mentioned antibodies. Western Blot analysis was carried out as described above.

### 2.8. ATP7B Complementation of HepG2 ATPB^−/−^ Cells

In a 100 mm cell culture dish, 1 × 10^6^ HepG2 ATP7B^−/−^ cells were transfected with 7.25 µg of the EGFP-ATP7B-WT or EGFP-ATP7B^W1452A/L1455A^ vectors using Lipofectamine™ LTX Reagent with PLUS™ Reagent (Thermo Fisher Scientific, 15338100). 24 h post-transfection, cells were treated overnight with varied concentrations of CuSO_4_. The next day, cells were trypsinized and collected for fluorescence-activated cell sorting (FACS). Positively transfected, EGFP-positive cells were separated from EGFP-negative cells and both fractions were prepared for western blot as above.

### 2.9. Colocalization Experiments

For the detection of the effect of copper on autophagosome-lysosome fusion, 50,000 cells of each HepG2 WT and HepG2 ATP7B^−/−^ were grown on laminin-coated coverslips for 24 h, transduced with BacMam-GFP-RFP-LC3B overnight according to the manufacturer’s recommendations. The cells were then treated with CuSO_4_ at a concentration range from 100 µM to 800 µM for 24 h, fixed with 4% paraformaldehyde for 15 min and finally stained with 250 ng/mL DAPI (Thermo Fisher scientific, Molecular Probes, D1306) for 5 min. The coverslips were fixed on a glass slide using Mowiol 4–88 (Sigma Aldrich, 81381). The fluorescence cell imaging was acquired using a confocal Zeiss LSM 780 microscope with a 63 × 1.40 Oil DIC M27. The image sections with a comparable technical quality of BacMam signal strength and antibodies staining signal were randomly selected under the microscope to ensure an unbiased analysis. Red and yellow puncta count per cell was done using the particle analysis in image J. For protein ATP7B trafficking analysis, 50,000 cells of HepG2 WT and HepG2 ATP7B^−/−^ were cultured in a 500 µL cell culture medium on a laminin-coated coverslip for 24 h, then transfected with EGFP-ATP7B. After 6 h post transfection, cells were transduced with BacMam-RFP-Golgi or the BacMam-RFP-Lysosome overnight, then treated with various concentrations of CuSO_4_ or 200 µM of bathocuproine disulfonate (BCS) for 24 h or EBSS for 2 h, respectively. To detect LC3B or endogenous ATP7B, the fixed cells were incubated with anti-LC3B or anti-ATP7B antibodies at 4 °C overnight followed by secondary antibody incubation, Alexa Fluor 568 goat anti-rabbit, at temperature for 1 h in the dark. Pearson’s correlation coefficient was analyzed per cell using the colocalization threshold of ImageJ Fiji.

### 2.10. Statistical Analysis

Statistical analyses were performed using two-way ANOVA followed by Sidak’s multiple comparisons test using GraphPad Prism 5.0. The variance and differences with *p* < 0.05 were considered significant.

## 3. Results

### 3.1. Investigation of LC3-ATP7B Interaction

**Figure 1:**
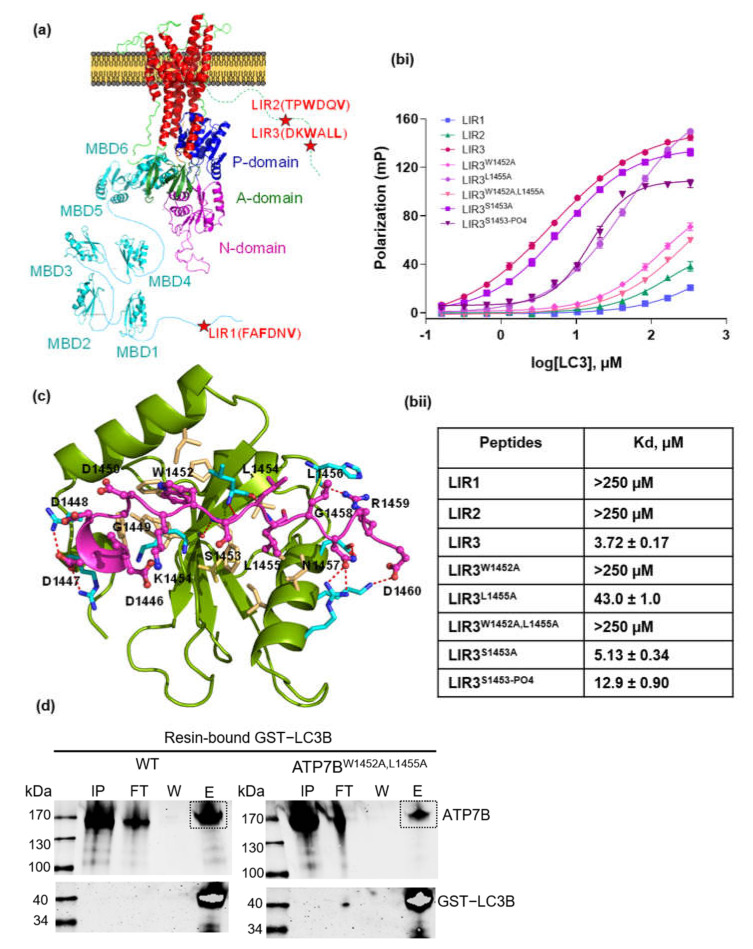
Detection of direct interaction between LC3B and ATP7B via LIR3. (**a**) Structural organization of ATP7B indicating the location of the three potential LIR peptides on ATP7B protein. (**bi**) Fluorescent polarization curves with a nonlinear curve fitting of the one site-specific binding. (**bii**) Quantification of *K*_d_ values from (**bi**), Experiments were done in three replications. (**c**) Protein peptide docking. The structural LC3B/LIR3-ATP7B complex was modeled on the crystal structure of the LC3B-LIR of SQSTM/p62 complex (PDB: 2ZJD) using GalaxyWEB server [30]. The LC3B protein is shown in green, the LIR3-ATP7B is shown in magenta. The hydrophobic residues are shown in yellow and the hydrogen bonds are indicated with the red dashed line. (**d**) Protein pull-down assay. The resin bound GST-LC3B protein was used as bait to pull WT and variant ATP7B^W1452A,L1455A^ from the cell lysate of transfected HEK293H. The pull-down experiment was repeated two times. The input, flow-through, final wash and elution fractions are indicated as IP, FT, W and E, respectively. ATP7B in the elution fraction is indicated with the black dashed box.

Several autophagy associated proteins contain functional LC3 interacting regions, so-called LIR motifs. The LIR motif consists of a conserved W/F/YxxL/I/V sequence, in which the conserved aromatic residues are the key residues involved in hydrophobic interactions with the LC3 protein [27,28]. We employed an iLIR online server to identify potential LIR motifs on the ATP7B protein [25]. The iLIR online server identified 21 of the conserved W/F/YxxL/I/V motifs on ATP7B (Supplementary Figure S1a). As most of these motifs are located on rigid areas of the protein structure, they are not likely to interact with LC3 [29]. Thus, further filtering was required and led to three potential LIRs located on flexible structural parts of ATP7B. The first motif, LIR1, containing the sequence of FAFDNV, is located on the flexible N-terminal region, while the second and third motifs, LIR2 and LIR3, containing sequence of TPWDQV and DKWSLL, respectively, are located on the flexible C-terminal region (Figure 1a).

To investigate direct physical interaction, fluorescently labeled peptides containing LIR1-3 sequences from ATP7B were tested for binding with purified LC3B protein using the fluorescence polarization assay. Under the tested conditions, LC3B bound to LIR1 and LIR2 with such low affinity (*K*_d_ > 250 µM) that actual binding under natural conditions is unlikely (Figure 1b). By contrast, LIR3 showed significant binding affinity with LC3B (*K*_d_ = 3.72 ± 0.17 µM). Molecular docking simulations revealed that W1452 and L1455 of LIR3 interact in the canonical way with the first and second hydrophobic region on LC3B, respectively (Figure 1c). In order to verify the importance of this interaction, residues W1452 and L1455 were substituted to alanine. W1452A substitution completely abolished LC3B interaction with no difference to the double substitution W1452A/L1455A (in both *K*_d_ > 250 µM), whereas L1455A showed a residual binding (*K*_d_ = 43.0 ± 1.0 µM), which was, however, decreased ninefold compared to native LIR3 (Figure 1b). Multiple sequence alignment of ATP7B among different species confirmed the LIR3 motif conservation (Supplementary Figure S1b). We also investigated the S1453 of LIR3, as it was found to be phosphorylated. S1453A substitution had no effect on the LC3B binding. However, the phosphorylation of S1453 caused a threefold increase in dissociation constant (*K*_d_ = 12.9 ± 0.9 µM) (Figure 1b).

Interestingly, the tryptophan 1452 in ATP7B was found to be replaced with Histidine residue in ATP7A which suggests that LIR3 may represent a unique property of ATP7B in cellular communication between the copper metabolism and the autophagy pathways. Finally a pull-down assay was used to confirm direct binding of full-length ATP7B to LC3B. Recombinant GST-LC3B expressed in *E. coli* BL21 cells was used as bait to pull recombinant ATP7B from a HEK293H cell homogenate. As expected, wild-type ATP7B protein was detected in the elution fraction (Figure 1d), whereas binding of the variant W1452A/L1455A (ATP7B^W1452A/L1455A^) was largely reduced. The technical control using GST only as bait was negative for ATP7B in the elution fraction (Supplementary Figure S2a). The decreased binding of recombinant ATP7B^W1452A/L1455A^ compared to WT was also observed in samples from HepG2 ATP7B^−/−^ cell homogenates (Supplementary Figure S2b).

### 3.2. Copper Induces Autophagy

**Figure 2:**
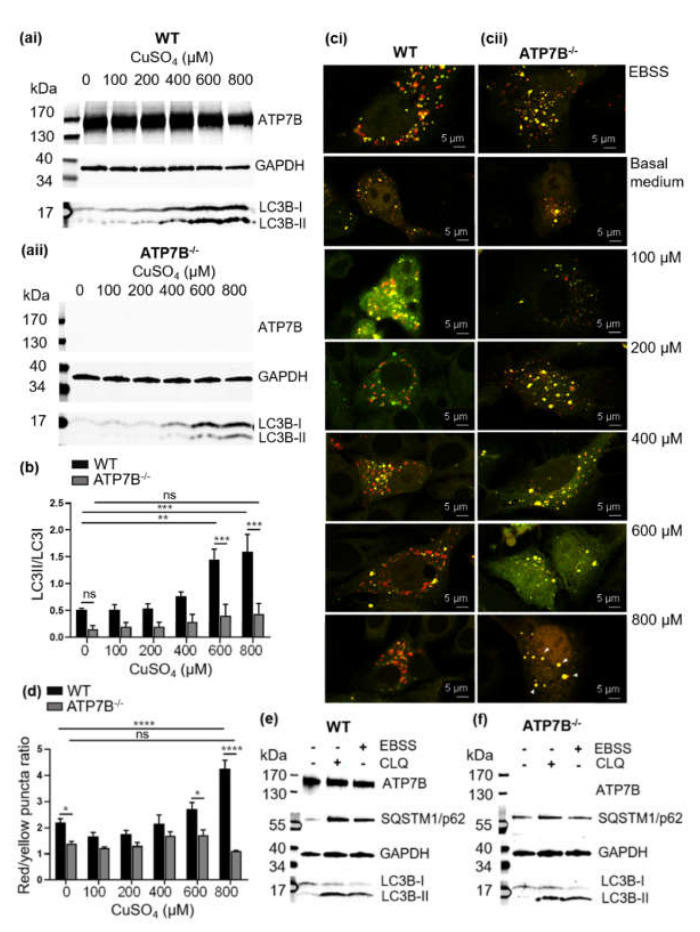
Course of copper-induced autophagy in HepG2 WT and HepG2 ATP7B^−/−^ cells. Western blot analysis of LC3B-II/LC3B-I ratio of copper-treated HepG2 WT (**ai**) and HepG2 ATP7B^−/−^ (**aii**) cells. (**b**) Quantification of western blot band intensity of LC3B-II/LC3B-I ratio. Autophagosome-lysosome fusion analysis in the HepG2 WT (**ci**) and HepG2 ATP7B^−/−^ cells (**cii**) under starvation (EBSS) and with copper added. The tandem GFP-RFP-LC3B was used as the indicator for autophagosome-lysosome fusion. Red puncta indicated fused autophagosome with lysosome and the yellow puncta indicated unfused autophagosome. (**d**) Analysis of red and yellow puncta ratio indicates the autophagosome-lysosome fusion efficiency. Autophagosome puncta were counted from 30–50 cells at each condition. Western blot analysis to identify the effect of starvation or chloroquine (CLQ) treated conditions in the HepG2 WT (**e**) and HepG2 ATP7B^−/−^ (**f**) cells. Statistical differences in the studied groups were assessed as follows: * *p* ≤ 0.05, ** *p* ≤ 0.01, *** *p* ≤ 0.001, **** *p* ≤ 0.0001 and ns = not significant.

Autophagy induction in the HepG2 WT cells was investigated following a 24 h treatment with CuSO_4_ at a concentration range of 100 µM to 800 µM. A significant increase of LC3B-II was observed at high CuSO_4_ concentrations of 600 µM and 800 µM (Figure 2 (ai),b). A control experiment with CuCl_2_ showed a similar result (Supplementary Figure S2c) indicating that different copper sources had no apparent influence on autophagy induction in HepG2 cells.

Next we wanted to check whether the increase of the LC3B-II is due to copper-induced autophagy in HepG2 cells and not a defect in autophagic flux due to a defective fusion of autophagosomes and lysosomes. We used a tandem sensor RFP-GFP-LC3B to monitor the autophagosome-lysosome fusion stage. An increased red/yellow puncta ratio occurred at a high CuSO_4_ concentration indicating acid-quenching of the GFP protein suggestive of the elevation of autophagosome-lysosome fusion (Figure 2(ci)). The trend of increased fusion was steady depending on CuSO_4_ concentration, and significance was reached at 800 µM.

CuSO_4_ (Figure 2d). Thus, autophagy was activated depending on the copper and proceeded normally in the HepG2 cells.

### 3.3. Impairment of Copper-Induced Autophagy in HepG2 Cells Deficient of the ATP7B Copper Transporter (HepG2 ATP7B^−/−^)

To investigate copper-dependent induction of autophagy in the absence of ATP7B, HepG2 ATP7B^−/−^ cells were treated with CuSO_4_ concentrations ranging from 100–800 µM. There was no difference in the level of LC3B-I compared to the wild-type cells (Figure 2(ai,aii)). However, in ATP7B^−/−^ cells the level of LC3B-II was significantly lower upon treatment with CuSO_4_, and even at 600 µM and 800 µM the LC3B-II/LC3B-I ratio did not significantly alter (Figure 2b). The autophagosome and lysosome fusion activity was further observed in both cell lines. In contrast to the HepG2 WT cells, we found an increasing number of yellow puncta at higher CuSO_4_ concentrations in the HepG2 ATP7B^−/−^ cells (Figure 2(cii)). The red/yellow puncta ratio was significantly decreased at 800 µM CuSO_4_ (Figure 2d). All of those results indicate a disturbed autophagosome-lysosome fusion activity in the HepG2 ATP7B^−/−^ cells. To check if this is not a general autophagy problem in HepG2 ATP7B^−/−^ cells, we applied starvation conditions using EBSS as a positive control for autophagy induction, and CLQ was applied to detect the LC3B flux at basal medium condition. Both HepG2 WT and HepG2 ATP7B^−/−^ cells showed similar LC3B-I/LC3B-II conversion and elevation of the SQSTM1/p62 protein (Figure 2e,f). Moreover, HepG2 ATP7B^−/−^ cells also exhibited the autophagosome- fusion activity under the EBSS treatment as similar to the wild-type cell (Figure 2(ci,cii)). Therefore, the abnormalities of the LC3B-I/LC3B-II conversion and autophagosome-lysosome fusion occurred only under the copper-induced autophagy in the HepG2 ATP7B^−/−^ cells.

To further prove that the autophagy defect was a direct consequence of the ATP7B deficiency, we transiently transfected the HepG2 ATP7B^−/−^ cells with wild type and mutant W1452A/L1455A N-terminal EGFP-tagged ATP7B plasmid vectors (EGFP-ATP7B-WT and EGFP-ATP7B^W1452A/L1455A^). For analysis, the cells were sorted by flow cytometry to separate the untransfected cells from the positively transfected ones. At baseline copper level, LC3B-I to LC3B-II conversion was already evident in all cells complemented with either EGFP-ATP7B-WT or EGFP-ATP7B^W1452A/L1455A^ (Supplementary Figure S3(ai,bi)). However, with rising concentrations of CuSO_4_ the conversion deficit became evident in the EGFP-ATP7B^W1452A/L1455A^ transfected compared to the EGFP-ATP7B-WT complemented cells, but still significantly higher than in the ATP7B-deficient control cell fraction (Supplementary Figure S3(aii,bii),c). Hence, positively EGFP-ATP7B-WT transfected cells showed a trend towards normalization of LC3I/LC3II conversion comparable to the HepG2 WT cells (compare Figure 2(ai) and Supplementary Figure S3(ai)).

### 3.4. Copper-Dependent Cellular Trafficking of ATP7B in HepG2 Cells

HepG2 WT cells expressing EGFP-ATP7B-WT were treated for 24 h with CuSO_4_ to investigate cellular ATP7B localization. Surprisingly, only 2.6% of ATP7B was found colocalized with TGN positive compartments with no additional application of CuSO_4_; this condition is hereafter called “basal medium condition” referring to: DMEM plus 10% fetal bovine serum plus 1% Penicillin-Streptomycin. However, a large proportion (68.7%) of ATP7B was colocalized with lysosomal LAMP1 protein (Supplementary Figure S4a–d). After treatment with 400 µM CuSO_4_, the colocalization of EGFP-ATP7B with lysosomal structures even increased about 1.2-fold (83.6%) while no TGN fraction was detectable. Endogenous ATP7B protein in HepG2 cells was probed and also found highly colocalized with lysosomes and there was a small portion of ATP7B localized with TGN (4.4%) at basal medium condition in agreement with earlier studies in HepG2 cells [4,31]. The lysosomal fraction of ATP7B could likewise be elevated upon CuSO_4_ treatment while the TGN fraction of ATP7B disappeared (Supplementary Figure S4e,f).

**Figure 3:**
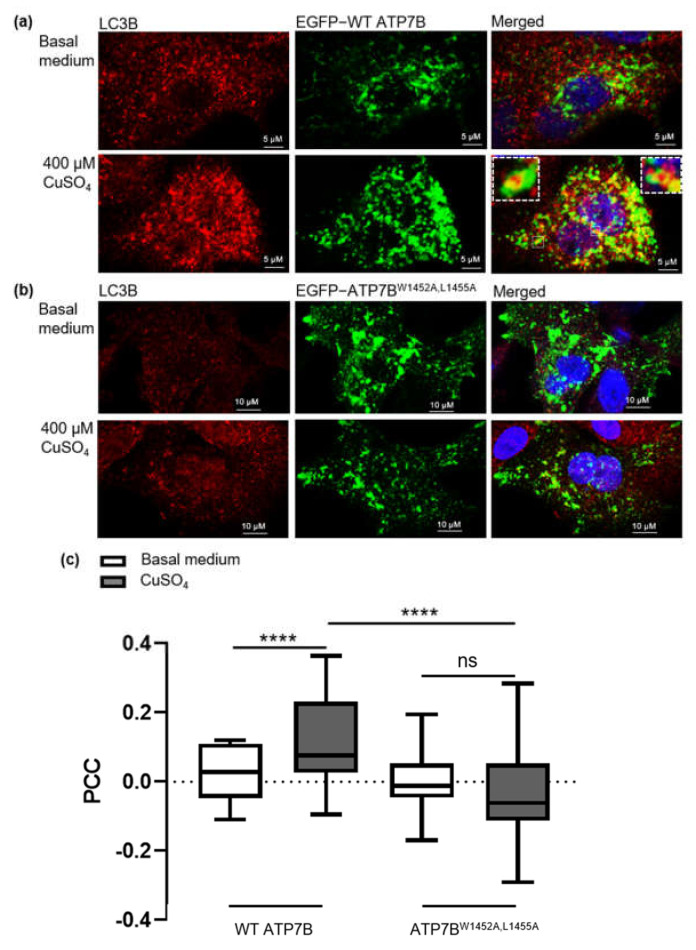
Colocalization of ATP7B wild-type and mutant ATP7B^W1452A,L1455A^ with LC3B. (**a**) Colocalization of EGFP-ATP7B wild-type with endogenous LC3B and (**b**) Colocalization of EGFP-ATP7B carrying the two LIR3 motif mutations W1452A and L1455A with endogenous LC3B. Experiments were done under basal medium condition and supplemented with 400 µM CuSO_4_. (**c**) Quantification of Pearson’s correlation coefficient from (**a**,**b**). Quantification was performed from counting 30 cells of each condition (**** *p* ≤ 0.0001 and ns = not significant).

As expected, only a small portion of EGFP-ATP7B was colocalized with the autophagy marker protein LC3B (2.4%) at basal copper concentration. This fraction was elevated under high CuSO_4_ concentration by 4.8-fold (11.6%) (Figure 3a,c and Supplementary Figure S4d). Moreover, the colocalization of the ATP7B and LC3B was particularly observed on the surface of autophagosome vesicles as shown in the inset of Figure 3a. ATP7B^W1452A/L1455A^ harboring the mutated LIR3 motif showed a similar preference to colocalize with LAMP1 positive compartments (77.8%) as the wild-type protein. However, it failed to colocalize with TGN under basal medium conditions (Supplementary Figure S5a,b) and showed inability to translocate to LC3B positive vesicles in response to CuSO_4_ treatment (Figure 3b,c) indicating that this variant cannot perform its distinct purpose on autophagosome membranes.

Lastly, we investigated whether the traces of detectable copper in the basal medium (<1 µmol/L) significantly altered ATP7B localization in the HepG2 cells. In fact, adding the copper chelator BCS to remove residual copper from the medium prior to the examination substantially elevated the ATP7B fraction in the TGN about 12.6-fold (32.8%) compared to basal medium condition (Supplementary Figure S4a,c,d). Despite the changed baseline situation with significantly more ATP7B in the TGN before CuSO_4_ treatment, the autophagosomal fraction of ATP7B was not elevated upon the addition of CuSO_4_ compared to ATP7B in cells under basal medium condition (Figure 3c). This suggests that the fraction of 11.6% LC3B colocalized ATP7B did not directly translocate from the TGN, warranting further research into the mechanism of ATP7B transport process under high copper levels.

## 4. Discussion

Several recent studies have examined the relationship between copper metabolism and autophagy. Some found that excess copper negatively influenced autophagy and impaired autophagic-lysosome degradation [8,32]. Recent research demonstrated that copper induced autophagy to protect cells from apoptotic cell death [9]. Our study demonstrated that copper induces autophagy in HepG2 WT cells. However, HepG2 ATP7B^−/−^ cells revealed an abnormal course of autophagy at the LC3B-I to LC3B-II conversion stage under excess CuSO_4_ overload >600 µM. A previous study demonstrated that oxidative stress-induction with H_2_O_2_ impaired the conversion of LC3B-I to LC3B-II due to dysfunctionalization of the ubiquitination-like proteins ATG3 and ATG7, which are crucial for LC3B-II formation [33]. Since H_2_O_2_ can be further decomposed into reactive oxygen species via Cu(I) involvement [34], this may serve as a valid explanation for the observed effects. Moreover, autophagosome-lysosome fusion was abnormal in HepG2 ATP7B^−/−^ cells. Damaged lysosomes may cause impaired autophagosome-lysosome fusion, as irregular-sized and -shaped lysosome structures have been previously observed in the HepG2 ATP7B^−/−^ cells under excess copper [9]. In order to closely investigate the autophagy dysfunction, we initially intended to monitor the degradation of SQSTM/p62 on western blot as well to demonstrate an altered autophagosome-lysosome fusion state in HepG2 ATP7B^−/−^. To our surprise, however, we observed the phenomenon that the SQSTM/p62 levels were subject to large interexperimental variation. A recent article reported that SQSTM/p62 gene expression can be stalled in HepG2 cells during prolonged starvation, which may lead to misinterpreted autophagy activation [35]. Therefore, an experimental alternative in the form of the Tandem GFP-RFP-LC3 was used to demonstrate the autophagosome-lysosome fusion state instead of SQSTM/p62. In any case, the presence of ATP7B is necessary to avoid cellular damage. The rescue of the LC3-I to LC3-II conversion phenotype in HepG2 ATP7B^−/−^ cells by episomal ATP7B expression further suggests the prerequisite of the presence of ATP7B for the proper functioning of copper-induced autophagy. An effect that occurs via the direct, rapid copper shuttling is conceivable due to the rapid kinetics of complementation.

We hypothesized that ATP7B recognized LC3B via its LIR motif and translocates to LC3B-positive compartments such as autophagosomes. ATP7B, disrupted of the LIR3 motif by mutating tryptophan 1452 and leucine 1455, failed to translocate to the autophagosome membrane. Moreover, the mutant also failed to normalize the LC3-I to LC3-II conversion phenotype, indicating a persisting autophagosome-lysosome fusion deficit in the HepG2 ATP7B^−/−^ cells under the excess copper condition. These results suggest that ATP7B–LC3B interaction and ATP7B translocation are important stages in the autophagy process under copper-elevated condition in the HepG2 cells. Further studies are required to investigate whether this interaction has a regulatory task to facilitate copper uptake into autophagosomes.

Only a small portion of ATP7B was found to be colocalized with LC3B in the HepG2 cells, either at basal medium condition or elevated copper. Instead, ATP7B is mainly localized on lysosomes in the HepG2 cells. Consistent with this finding, lysosomal localization of ATP7B in HepG2 cells at both low and high copper conditions was reported previously [4,36]. Other cell lines of hepatocytic origin, e.g., WIF-B and Can10, have been described as lacking the association of ATP7B with the lysosome at basal copper conditions, but show lysosomal translocation under high copper concentrations [37,38]. These cells may be more appropriate to study the ATP7B transport path beginning in the TGN.

A biochemical interpretation of the higher abundance of ATP7B on LAMP1 instead of LC3B-positive compartments could be that the interaction of the ATP7B with lysosomal targeting proteins might be stronger than the interaction with LC3B. This is due to the fact that ATP7B contains the conserved [D/E]xxx[LL] motif required for interaction with clathrin-associated adaptor protein complexes (APs), which target proteins on the clathrin coated vesicles to the lysosomes [39]. Remarkably, the [D/E]xxx[LL] motif of ATP7B, which is required for AP interaction, is identical to the amino acid sequence of LIR3 (DKWSLL), which is required for LC3B interaction. It is important to note that the hydrophobic residues W1452 and L1455 that are required for the LC3B interaction are also crucial for ATP7B localization at the TGN, as the double mutation abolished the TGN localization while localization with lysosome remained similar to the wild-type as shown in Supplementary Figures S4a,b and S5a,b. This result suggests that residues W1452 and/or L1455 are important for the localization on the TGN [40,41] and that APs, likely AP1, are involved in the ATP7B localization on the TGN and retrieval from lysosomes since mutations of L1454 and L1455 on ATP7B significantly reduced interaction with AP1 [41]. However, the substitutions of either W1452 and L1455 with Alanine had no effect on lysosomal localization (suggesting that sorting of ATP7B to lysosomes emerges in an AP independent manner), but rather uses a Golgi bypassing route to cytoplasmic vesicles such as endosomes, autophagosomes and lysosomes directly from ER [42].

As the interaction of ATP7B with LC3B or the AP proteins is at the same site, there should be a molecular switch to define the interaction. It has been previously reported that the hyperphosphorylated ATP7B under the copper elevated condition facilitated ATP7B trafficking to cytoplasmic vesicles [43,44]. The S1453 within the DKWSLL motif was one of the residues found to be phosphorylated. Therefore, we further investigated the effect of the phosphorylation of the S1453 on the interaction with LC3B. We found a threefold increase in the dissociation constant, indicating that the phosphorylation status of LIR3 residue S1453 is likely to play a role in LC3B binding.

## 5. Conclusions

These high copper concentrations can trigger autophagy in the cell. This study provides the first evidence of a direct physical interaction between the LIR3 motif of the ATP7B copper transporting ATPase and the *bona fide* marker of autophagy, LC3B. More specifically, ATP7B localization on autophagosomal compartments was observed upon CuSO_4_ treatment in HepG2 WT cells and ATP7B, recruited to the autophagosome membrane, may in turn facilitate the copper uptake into the autophagosome. Internalized copper may subsequently be eliminated into the extracellular space for further disposal. The abnormal autophagy process in HepG2-ATP7B^−/−^ cells is evidence that the loss of ATP7B prevents the important interaction between ATP7B and LC3B at the interface between copper metabolism and the autophagy pathway. Moreover, the impaired autophagosome-lysosome fusion in the HepG2 ATP7B^−/−^ cell suggests an alternative, more complex role of ATP7B on the clearance of excess copper involving regulated intracellular translocation and facilitation of the autophagosome-lysosome fusion process at copper-induced autophagy, but the exact role of ATP7B in this context remains elusive.

## Data Availability

Data is contained within the article and Supplementary Materials.

## Supplementary Materials

The following are available online at https://www.mdpi.com/article/10.3390/cells10113118/s1, Supplementary Figure S1: Identification of LIR motifs on ATP7B, Supplementary Figure S2: Role of ATP7B on autophagy in HepG2 WT and HepG2 ATP7B−/− cells, Supplementary Figure S3: Observation of autophagy activity in EGFP-ATP7B-WT and EGFP-ATP7BW1542A/L1455A transfected HepG2 ATP7B−/− cells, Supplementary Figure S4: Localization analysis of ATP7B in HepG2 cells, Supplementary Figure S5: Colocalization of mutant ATP7BW1452A,L1455A with TGN and lysosomes, Supplementary Table S1: Sequences and monoisotopic masses of the peptides used in the study.

## References

[1] Camakaris J., Voskoboinik I., Mercer J.F. (1999). Molecular mechanisms of copper homeostasis. Biochem. Biophys. Res. Commun..

[2] Lutsenko S. (2010). Human copper homeostasis: A network of interconnected pathways. Curr. Opin. Chem. Biol..

[3] Blaby-Haas C.E., Merchant S.S. (2014). Lysosome-related organelles as mediators of metal homeostasis. J. Biol. Chem..

[4] Polishchuk E.V., Concilli M., Iacobacci S., Chesi G., Pastore N., Piccolo P., Paladino S., Baldantoni D., van IJzendoorn S.C.D., Chan J. (2014). Wilson disease protein ATP7B utilizes lysosomal exocytosis to maintain copper homeostasis. Dev. Cell.

[5] Gupta A., Lutsenko S. (2009). Human copper transporters: Mechanism, role in human diseases and therapeutic potential. Future Med. Chem..

[6] Bartee M.Y., Lutsenko S. (2007). Hepatic copper-transporting ATPase ATP7B: Function and inactivation at the molecular and cellular level. Biometals.

[7] Hellman N.E., Kono S., Mancini G.M., Hoogeboom A.J., de Jong G.J., Gitlin J.D. (2002). Mechanisms of copper incorporation into human ceruloplasmin. J. Biol. Chem..

[8] Masaldan S., Clatworthy S.A.S., Gamell C., Smith Z.M., Francis P.S., Denoyer D., Meggyesy P.M., La Fontaine S., Cater M.A. (2018). Copper accumulation in senescent cells: Interplay between copper transporters and impaired autophagy. Redox Biol..

[9] Polishchuk E.V., Merolla A., Lichtmannegger J., Romano A., Indrieri A., Ilyechova E.Y., Concilli M., de Cegli R., Crispino R., Mariniello M. (2019). Activation of Autophagy, Observed in Liver Tissues from Patients with Wilson Disease and from ATP7B-Deficient Animals, Protects Hepatocytes from Copper-Induced Apoptosis. Gastroenterology.

[10] Stolz A., Ernst A., Dikic I. (2014). Cargo recognition and trafficking in selective autophagy. Nat. Cell Biol..

[11] Mizushima N., Levine B., Cuervo A.M., Klionsky D.J. (2008). Autophagy fights disease through cellular self-digestion. Nature.

[12] Lamb C.A., Yoshimori T., Tooze S.A. (2013). The autophagosome: Origins unknown, biogenesis complex. Nat. Rev. Mol. Cell Biol..

[13] Xie Z., Nair U., Klionsky D.J. (2008). Dissecting autophagosome formation: The missing pieces. Autophagy.

[14] Sou Y.-s., Waguri S., Iwata J.-i., Ueno T., Fujimura T., Hara T., Sawada N., Yamada A., Mizushima N., Uchiyama Y. (2008). The Atg8 conjugation system is indispensable for proper development of autophagic isolation membranes in mice. Mol. Biol. Cell.

[15] Kirisako T., Ichimura Y., Okada H., Kabeya Y., Mizushima N., Yoshimori T., Ohsumi M., Takao T., Noda T., Ohsumi Y. (2000). The reversible modification regulates the membrane-binding state of Apg8/Aut7 essential for autophagy and the cytoplasm to vacuole targeting pathway. J. Cell Biol..

[16] Pankiv S., Clausen T.H., Lamark T., Brech A., Bruun J.-A., Outzen H., Øvervatn A., Bjørkøy G., Johansen T. (2007). p62/SQSTM1 binds directly to Atg8/LC3 to facilitate degradation of ubiquitinated protein aggregates by autophagy. J. Biol. Chem..

[17] Kirkin V., McEwan D.G., Novak I., Dikic I. (2009). A role for ubiquitin in selective autophagy. Mol. Cell.

[18] Zheng Y.T., Shahnazari S., Brech A., Lamark T., Johansen T., Brumell J.H. (2009). The adaptor protein p62/SQSTM1 targets invading bacteria to the autophagy pathway. J. Immunol..

[19] Von Muhlinen N., Akutsu M., Ravenhill B.J., Foeglein Á., Bloor S., Rutherford T.J., Freund S.M.V., Komander D., Randow F. (2012). LC3C, bound selectively by a noncanonical LIR motif in NDP52, is required for antibacterial autophagy. Mol. Cell.

[20] Johansen T., Lamark T. (2020). Selective Autophagy: ATG8 Family Proteins, LIR Motifs and Cargo Receptors. J. Mol. Biol..

[21] Satoo K., Noda N.N., Kumeta H., Fujioka Y., Mizushima N., Ohsumi Y., Inagaki F. (2009). The structure of Atg4B-LC3 complex reveals the mechanism of LC3 processing and delipidation during autophagy. EMBO J..

[22] Alemu E.A., Lamark T., Torgersen K.M., Birgisdottir A.B., Larsen K.B., Jain A., Olsvik H., Øvervatn A., Kirkin V., Johansen T. (2012). ATG8 family proteins act as scaffolds for assembly of the ULK complex: Sequence requirements for LC3-interacting region (LIR) motifs. J. Biol. Chem..

[23] Yang A., Pantoom S., Wu Y.-W. (2017). Elucidation of the anti-autophagy mechanism of the Legionella effector RavZ using semisynthetic LC3 proteins. Elife.

[24] Pantoom S., Yang A., Wu Y.-W. (2017). Lift and cut: Anti-host autophagy mechanism of Legionella pneumophila. Autophagy.

[25] Kalvari I., Tsompanis S., Mulakkal N.C., Osgood R., Johansen T., Nezis I.P., Promponas V.J. (2014). iLIR: A web resource for prediction of Atg8-family interacting proteins. Autophagy.

[26] Bohl C., Pomorski A., Seemann S., Knospe A.-M., Zheng C., Krężel A., Rolfs A., Lukas J. (2017). Fluorescent probes for selective protein labeling in lysosomes: A case of α-galactosidase A. FASEB J..

[27] Noda N.N., Kumeta H., Nakatogawa H., Satoo K., Adachi W., Ishii J., Fujioka Y., Ohsumi Y., Inagaki F. (2008). Structural basis of target recognition by Atg8/LC3 during selective autophagy. Genes Cells.

[28] Ichimura Y., Kumanomidou T., Sou Y.-s., Mizushima T., Ezaki J., Ueno T., Kominami E., Yamane T., Tanaka K., Komatsu M. (2008). Structural basis for sorting mechanism of p62 in selective autophagy. J. Biol. Chem..

[29] Popelka H., Klionsky D.J. (2015). Analysis of the native conformation of the LIR/AIM motif in the Atg8/LC3/GABARAP-binding proteins. Autophagy.

[30] Lee H., Heo L., Lee M.S., Seok C. (2015). GalaxyPepDock: A protein-peptide docking tool based on interaction similarity and energy optimization. Nucleic Acids Res..

[31] Harada M., Sakisaka S., Terada K., Kimura R., Kawaguchi T., Koga H., Taniguchi E., Sasatomi K., Miura N., Suganuma T. (2000). Role of ATP7B in biliary copper excretion in a human hepatoma cell line and normal rat hepatocytes. Gastroenterology.

[32] Tan X., Guan H., Yang Y., Luo S., Hou L., Chen H., Li J. (2020). Cu(II) disrupts autophagy-mediated lysosomal degradation of oligomeric Aβ in microglia via mTOR-TFEB pathway. Toxicol. Appl. Pharmacol..

[33] Frudd K., Burgoyne T., Burgoyne J.R. (2018). Oxidation of Atg3 and Atg7 mediates inhibition of autophagy. Nat. Commun..

[34] Li W., Zhou P., Zhang J., Zhang Y., Zhang G., Liu Y., Cheng X. (2018). Generation of reactive oxygen species by promoting the Cu(II)/Cu(I) redox cycle with reducing agents in aerobic aqueous solution. Water Sci. Technol..

[35] Sahani M.H., Itakura E., Mizushima N. (2014). Expression of the autophagy substrate SQSTM1/p62 is restored during prolonged starvation depending on transcriptional upregulation and autophagy-derived amino acids. Autophagy.

[36] Polishchuk E.V., Polishchuk R.S. (2016). The emerging role of lysosomes in copper homeostasis. Metallomics.

[37] Polishchuk R.S., Polishchuk E.V. (2019). From and to the Golgi—Defining the Wilson disease protein road map. FEBS Lett..

[38] Nyasae L.K., Schell M.J., Hubbard A.L. (2014). Copper directs ATP7B to the apical domain of hepatic cells via basolateral endosomes. Traffic.

[39] Braulke T., Bonifacino J.S. (2009). Sorting of lysosomal proteins. Biochim. Biophys. Acta.

[40] Lalioti V., Hernandez-Tiedra S., Sandoval I.V. (2014). DKWSLLL, a versatile DXXXLL-type signal with distinct roles in the Cu(+)-regulated trafficking of ATP7B. Traffic.

[41] Jain S., Farías G.G., Bonifacino J.S. (2015). Polarized sorting of the copper transporter ATP7B in neurons mediated by recognition of a dileucine signal by AP-1. Mol. Biol. Cell.

[42] Staudt C., Puissant E., Boonen M. (2016). Subcellular Trafficking of Mammalian Lysosomal Proteins: An Extended View. Int. J. Mol. Sci..

[43] Braiterman L.T., Gupta A., Chaerkady R., Cole R.N., Hubbard A.L. (2015). Communication between the N and C termini is required for copper-stimulated Ser/Thr phosphorylation of Cu(I)-ATPase (ATP7B). J. Biol. Chem..

[44] Pilankatta R., Lewis D., Adams C.M., Inesi G. (2009). High yield heterologous expression of wild-type and mutant Cu+-ATPase (ATP7B, Wilson disease protein) for functional characterization of catalytic activity and serine residues undergoing copper-dependent phosphorylation. J. Biol. Chem..

