# Supplementary material for: Direct Interaction of ATP7B and LC3B Proteins Suggests a Cooperative Role of Copper Transportation and Autophagy
Source: Cells. 2021 Nov 10;10(11):3118. doi: 10.3390/cells10113118 (PMC8625360; doi:10.3390/cells10113118)
Supplement: Supplementary file 1 [file cells-10-03118-s001.zip › cells-1432251-supplementary.pdf]

Supplementary Information

Direct interaction of ATP7B and LC3B proteins suggests a cooperative role of copper transportation and autophagy

Supansa Pantoom[a], Adam Pomorski[b], Katharina Huth[a], Christina Hund[a], Janine Petters[a], Artur Krężel[a], Andreas Hermann[a,c,d] and Jan Lukas[a,c*]

[a]Translational Neurodegeneration Section "Albrecht-Kossel", Department of Neurology, University Medical Center Rostock, 18147 Rostock, Germany
[b]Department of Chemical Biology, Faculty of Biotechnology, University of Wrocław, 50-383 Wrocław, Poland
[c]Center for Transdisciplinary Neurosciences Rostock (CTNR), University Medical Center Rostock, University of Rostock, 18147 Rostock, Germany
[d]German Center for Neurodegenerative Diseases (DZNE) Rostock/Greifswald, 18147 Rostock, Germany
* Correspondence: jan.lukas@med.uni-rostock.de

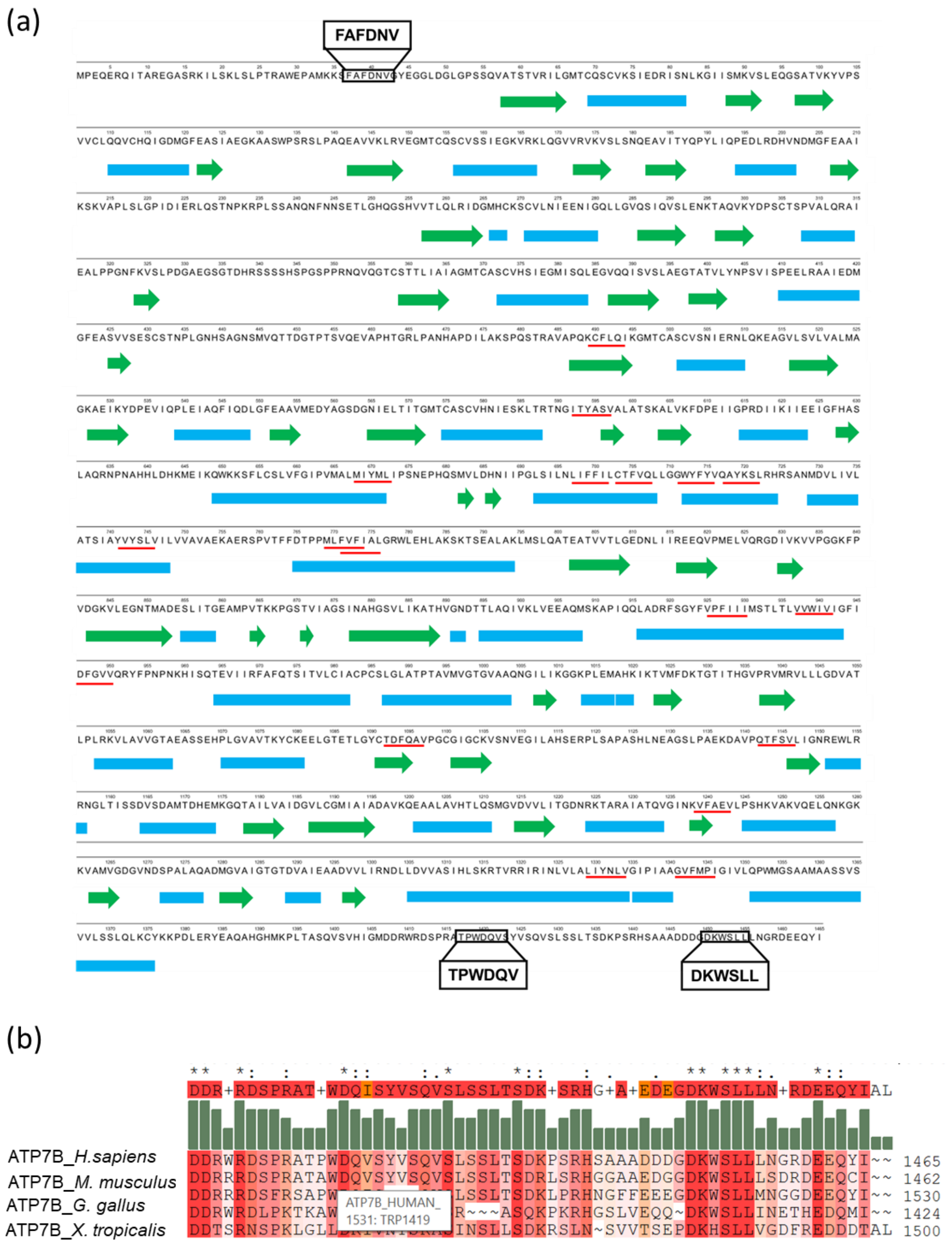

Supplementary Figure S1. Identification of LIR motifs on ATP7B. (a) Primary amino acid sequence and secondary structures of ATP7B. The location of secondary structures of ATP7B is indicated on the corresponding amino acid sequence based on the homology modeling structure of human ATP7B. Homology modeling structure of human ATP7B was built based on the crystal structure of *Legionella* ATP7B as a template (PDB: 4BBJ). Green arrows indicate the beta-barrel structure and blue boxes indicate helical structure. The three potential LIR motifs are specifically highlighted by the black boxes and another 18 canonical LIR-motifs located on the rigid structure of ATP7B are indicated as red lines. (b) Sequences of ATP7B from human (*H. sapiens*), mouse (*M. musculus*), chicken (*G. gallus*), western clawed frog (*X. tropicalis*) and ATP7A from human were aligned using Multiple Sequence Viewer (Schrödinger LLC, Mannheim, Germany). The residues are shaded based on their levels of conservation in the alignment.

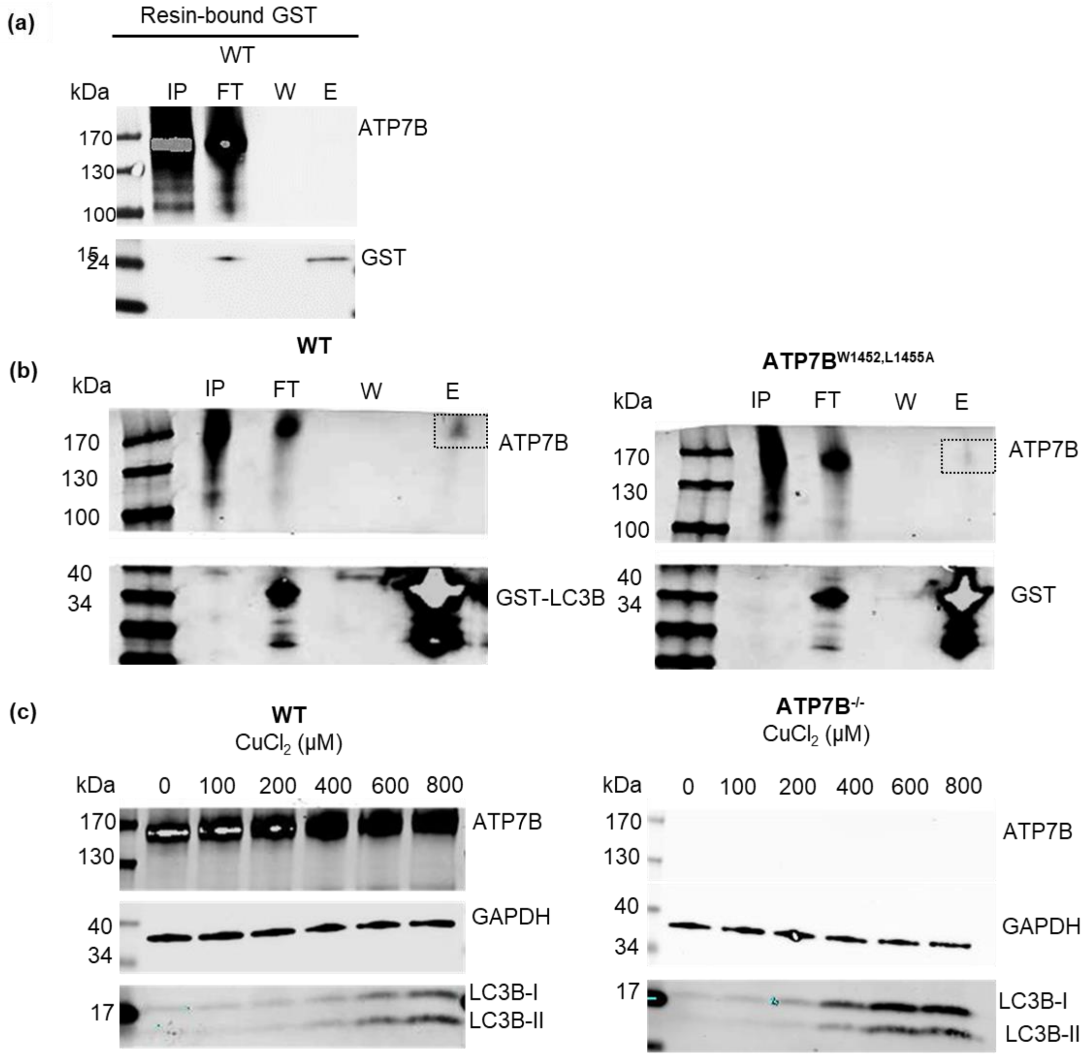

Supplementary Figure S2. Role of ATP7B on autophagy in HepG2 WT and HepG2 ATP7B$^{-/-}$ cells. (a) The negative control for protein pull-down assay. The resin-bound GST was used as a negative control to pull WT-ATP7B from the cell lysate of transfected HEK293H. ATP7B was detected with ATP7B antibody (Abcam, ab124973) and GST were detected with a GST antibody (Sigma-Aldrich, G7781). (b) ATP7B pull-down assay in HepG2 ATP7B$^{-/-}$ cells transfected with ATP7B WT and mutant ATP7B^{W1452A,L1455A} using resin bound GST-LC3B protein ATP7B was detected with ATP7B antibody while the GST-LC3B was detected with LC3B antibody. The input, flow-through, final wash and elution fractions are indicated as IP, FT, W and E respectively. ATP7B in the elution fraction is indicated with the black dashed box. (c) Western blot analysis of LC3B-I and LC3B-II after $CuCl_2$ treatment of HepG2 WT and HepG2 ATP7B$^{-/-}$ cells.

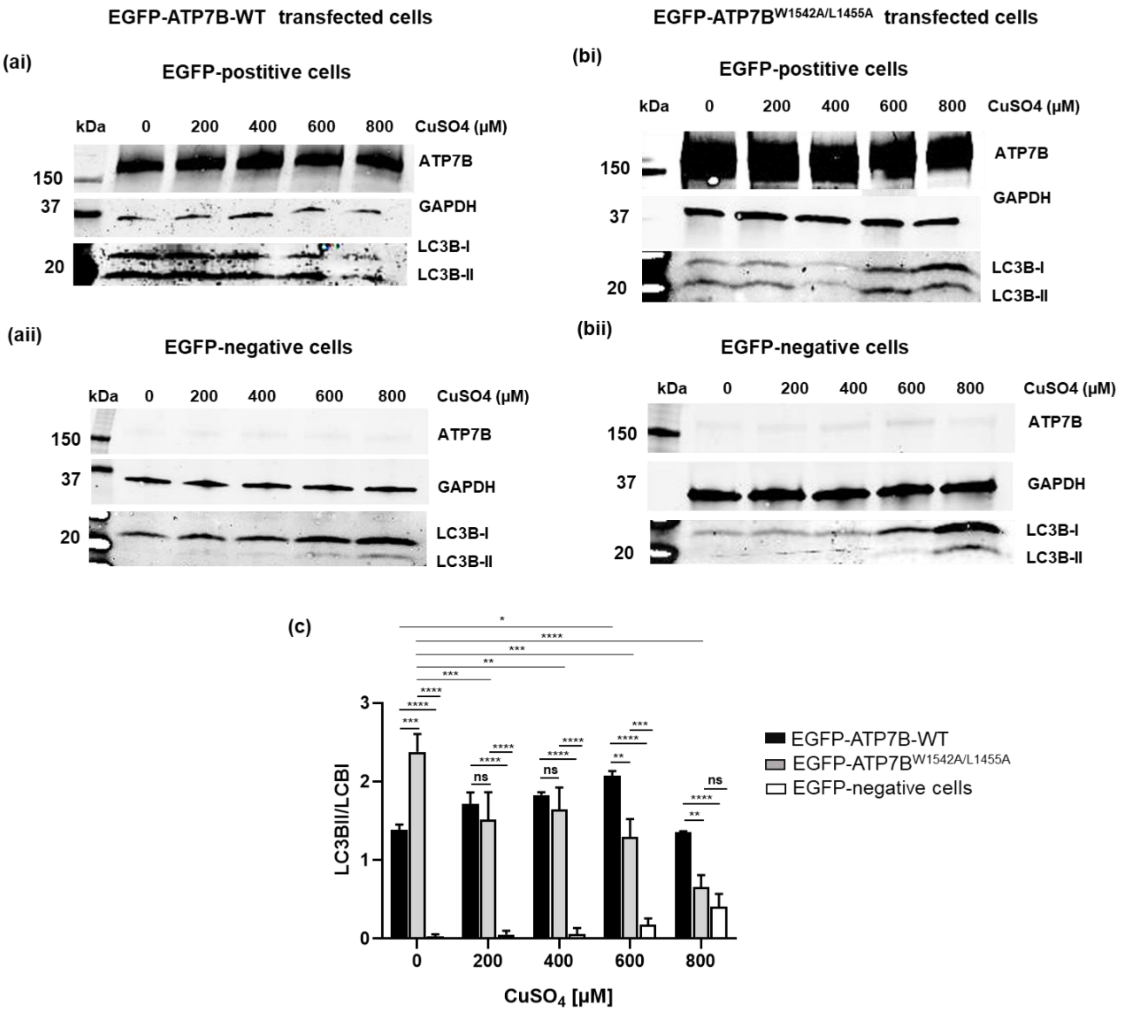

Supplementary Figure S3. Observation of autophagy activity in EGFP-ATP7B-WT and EGFP-ATP7B$^{W1542A/L1455A}$ transfected HepG2 ATP7B$^{-/-}$ cells. After transient transfection, positively and negatively transfected cells were sorted by EGFP fluo-rescence via FACS analysis and prepared for further analysis by western blot.Western blot analysis of EGFP-ATP7B-WT transfected HepG2 ATP7B$^{-/-}$ cells (A) and (B) the corresponding negative cell fraction(C) Western blot analysis of EGFP-ATP7B $^{W1542A/L1455A}$ transfected HepG2 ATP7B$^{-/-}$ cells (C) and the corresponding negative cell fraction. EGFP-ATP7B was detected with ATP7B antibody (Abcam, ab124973), the endogenous LC3B was detected with LC3B antibody (Cell Signal-ing Technology, 2775s) and GAPDH was detected with GAPDH antibody (abcam, ab8245) as a housekeeping protein. (E) Quantification of western blot band intensity of LC3B-II/LC3B-I ratio. The experiment was repeated two times. (* $p \leq 0.05$, ** $p \leq 0.01$, ***$p \leq 0.001$, ****$p \leq 0.0001$ and ns = not significant).

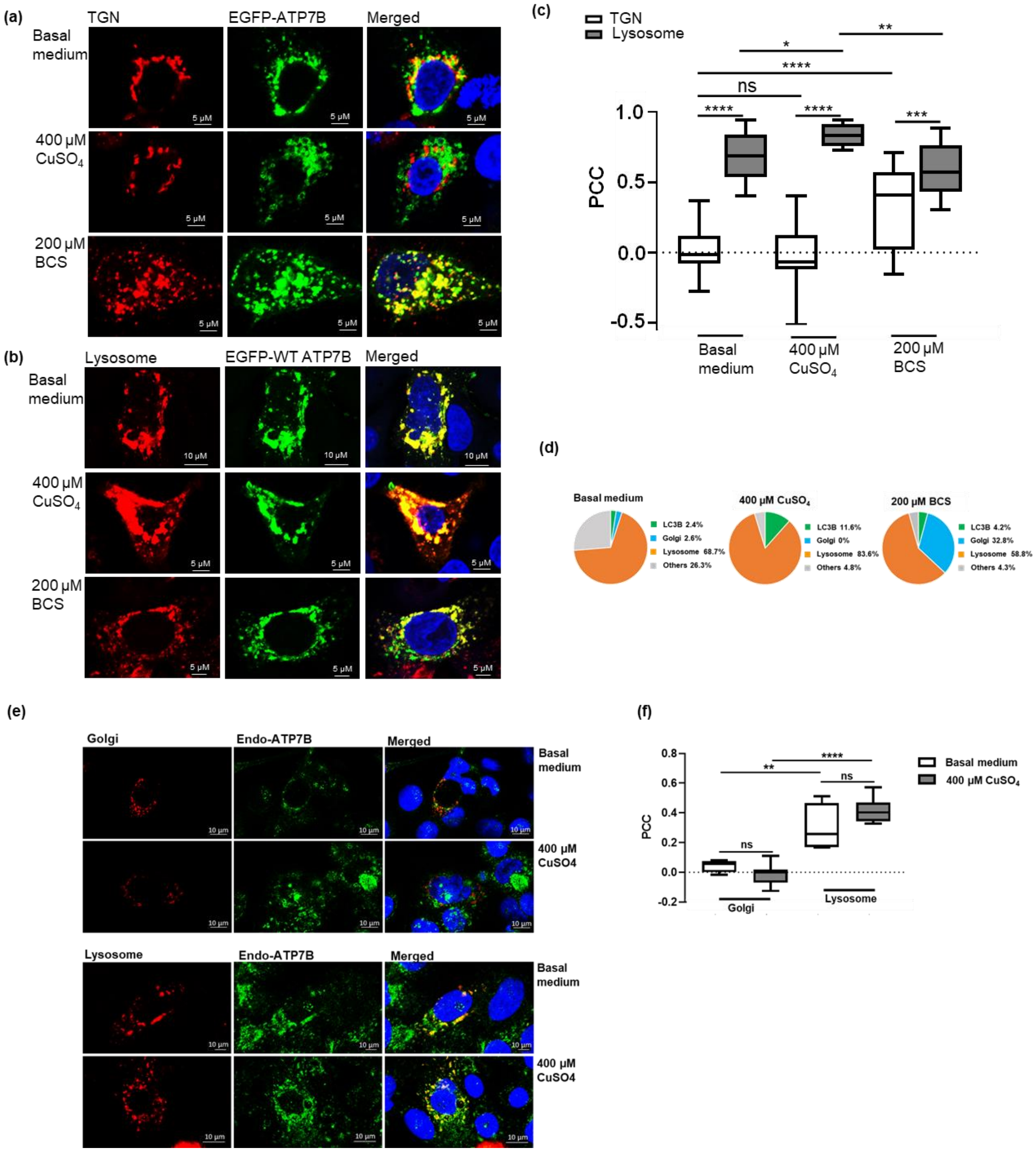

Supplementary Figure S4. Localization analysis of ATP7B in HepG2 cells. (a) EGFP-ATP7B WT was tracked in HepG2 cells with the BacMam RFP-N acetylglucosaminyltransferase for TGN localization. (b) Wild-type EGFP-ATP7B was tracked with BacMam, RFP-LAMP1 for lysosome localization. Experiments were done under basal medium condition or supple-mented with 400 μM $CuSO_4$ or 200 μM BCS, respectively. (c) Quantification of Pearson's correlation coefficient from (a) and (b). Quantification was performed from counting 30 cells of each condition (* $p \leq 0.05$, ** $p \leq 0.01$, ***$p \leq 0.001$, ****$p \leq 0.0001$ and ns = not significant). (d) Percentage of the colocalization of EGFP-WT ATP7B with cellular markers N-acetyl-galactosaminyltransferase (TGN), LAMP1 (lysosome) and LC3B. The percentage of the localization was calculated from the mean of the PCC with the respective colocalization partner. (e) Localization of endogenous ATP7B (Endo-ATP7B) was tracked with TGN and lysosome marker under either basal medium condition or 400 μM $CuSO_4$ treatment. (f) Quantifi-cation of Pearson's correlation coefficient of Endo-ATP7B with each cellular marker under basal medium condition or 400 μM $CuSO_4$ treatment (** $p \leq 0.01$, ****$p \leq 0.0001$ and ns = not significant). .

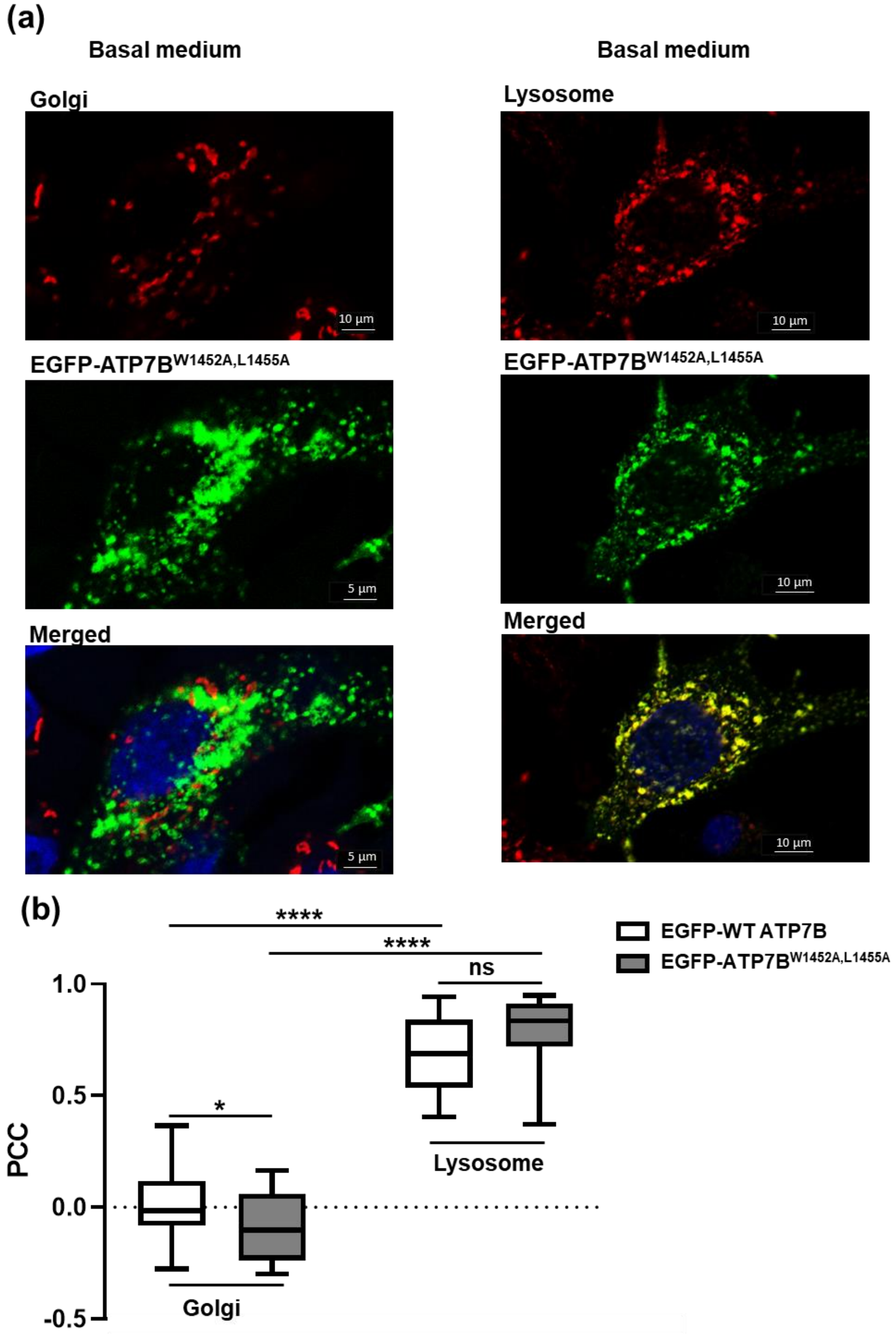

Supplementary Figure S5. Colocalization of mutant ATP7B^{W1452A,L1455A} with TGN and lysosomes. (a) EGFP-ATP7B^{W1452A,L1455A} was tracked with TGN and lysosome markers under basal medium condition. (b) Quantification of Pearson's correlation coefficient of mutant ATP7B^{W1452A,L1455A} with each cellular marker under basal medium condition (*$p \le 0.05$ and ****$p \le 0.0001$). .

Supplementary Table S1. Sequences and monoisotopic masses of the peptides used in the study. CF stands for 5-carboxyfluorescein.

Name	Sequence	Calculated monoisotopic mass	Experimental monoisotopic mass
LIR1	CF-MKKSFAFDNVGYEGGLD-amide	2234.2	2235.0
LIR2	CF-DSPRATPWDQVSYVSQ-amide	2192.2	2193.2
LIR3	CF-ADDDGDKWSLLLNGRD-amide	2146.2	2145.2
$LIR3^{W1452A}$	CF-ADDDGDKASLLLNGRD-amide	2031.1	2031.6
$LIR3^{L1455A}$	CF-ADDDGDKWSLALNGRD-amide	2104.1	2103.5
$LIR3^{W1452A,L1455A}$	CF-ADDDGDKASLALNGRD-amide	1989.1	1989.6
$LIR3^{S1453A}$	CF-ADDDGDKWALLLNGRD-amide	2130.2	2131.1
$LIR3^{S1453\text{-}PO4}$	CF-ADDDGDKWS(PO_4)LLLNGRD-amide	2226.2	2227.0

Note: In all cases the experimental monoisotopic mass was obtained by applying charge deconvolution.
